# Colored Indicator Undergloves Increase the Detection of Glove Perforations by Surgeons During Small Animal Orthopedic Surgery: A Randomized Controlled Trial

**DOI:** 10.1111/vsu.12519

**Published:** 2016-07-14

**Authors:** Lee B. Meakin, Oliver P. Gilman, Kevin J. Parsons, Neil J. Burton, Sorrel J. Langley‐Hobbs

**Affiliations:** ^1^ School of Veterinary ScienceUniversity of BristolBristolUnited Kingdom

## Abstract

**Objective:**

To determine whether use of colored indicator gloves affects perforation detection rate and to identify risk factors for glove perforation during veterinary orthopedic surgery.

**Study Design:**

Prospective randomized controlled trial.

**Sample Population:**

574 double pairs of gloves worn during 300 orthopedic surgical procedures (2,296 gloves).

**Methods:**

Primary and assistant surgeons double‐gloved for all orthopedic surgical procedures. Type of inner glove (standard or colored indicator) was randomized for the first 360 double pairs of gloves worn by surgeons during 180 procedures. Perforations detected by surgeons were recorded and gloves changed if requested. For a further 120 procedures, indicator gloves were used exclusively. All gloves were leak‐tested after surgery to identify perforations. Association between potential risk factors and perforation was explored using multivariate logistical regression analysis.

**Results:**

Glove perforations occurred during 43% of surgeries with a mean of 2.3 holes/surgery. Inner gloves were intact in 63% of glove pairs where an outer perforation occurred. Intraoperative perforation detection was improved when colored indicator gloves were worn (83% sensitivity) vs. standard gloves (34% sensitivity; *P*<.001). Independent risk factors for perforation were placement of plates and/or screws (*P*=.001; OR=2.4; 95% CI, 1.4–4.0), placement of an external skeletal fixator (*P*=.002; OR=7.0; 95% CI, 2.1–23.8), use of orthopedic wire (*P*=.011; OR=2.4; 95% CI, 1.2–4.7), and primary surgeon being board‐certified (*P*=.016; OR=1.9; 95% CI, 1.1–3.1).

**Conclusion:**

Increased surgeon recognition of glove perforations through use of colored indicator gloves enables prompt change of gloves if perforation occurs and may reduce potential contamination of the surgical site.

Surgical site infections (SSI) are an important concern in veterinary surgery, particularly with the increasing prevalence of multidrug resistant bacteria.^1^, ^2^, ^3^ To reduce the risk of postoperative SSI, strict adherence to Halstead's surgical principles is recommended,^4^ including the use of aseptic technique to limit iatrogenic contamination of the surgical site. One aspect of maintaining aseptic technique is the wearing of sterile operating gloves by the surgical team. However, glove perforations may occur during surgery, particularly when using sharp surgical instruments. This will reduce the gloves' effectiveness in providing an aseptic barrier leading to the potential for cross contamination from the surgeon to the patient and vice versa. Bacterial contamination of surgical gloves was reported to occur during 31% of procedures within a veterinary teaching hospital^5^ and glove perforations have recently been reported to occur during 26% of surgical procedures.^6^ The latter study also documented that glove perforations were observed approximately two times more frequently during orthopedic surgical procedures with the use of air‐powered tools and orthopedic wire being independent risk factors for glove perforation. However, risk factors for other orthopedic instrumentation were not investigated. An additional veterinary study also identified orthopedic surgery as a risk factor for glove perforation and documented defects in 32% of procedures compared with 19% of soft tissue procedures.^7^

The increased incidence of glove perforations in orthopedic surgery is concerning since a human study reported that glove perforation doubles the risk of SSI.^8^ Furthermore, SSI in the presence of permanent metallic orthopedic implants can lead to biofilm formation, reducing the effectiveness of systemic antibiotic therapy.^9^ For example, 30% of SSI after tibial plateau leveling osteotomy surgery required explantation of the plate and screws to resolve the infection.^10^ Repeated surgery increases morbidity and inflicts a financial burden on owners.^11^ In an attempt to provide an additional barrier, the use of double or triple gloving and knitted or steel outer gloves in human surgery has been assessed in a Cochrane meta‐analysis.^12^ This review concluded that the addition of a second pair of gloves reduced the perforation rate to inner gloves. However, to the authors' knowledge, the use of double gloving has to date not been evaluated in a veterinary study.

Despite the high incidence of glove perforation in a veterinary setting, the detection of glove perforations by surgeons is relatively poor with a sensitivity of 7–31%.^6^, ^7^ The higher of these detection rates is similar to that reported during human surgery (37%).^12^ One method employed to increase the detection of perforations by surgeons during procedures is the wearing of colored indicator gloves as the inner pair when double‐gloving.^13^ Indicator gloves are manufactured from standard surgical glove materials but have the addition of a colored dye. Should a perforation occur in the outer glove, fluid will leak through the breach in the outer glove causing a colored spot to appear, alerting the wearer to the perforation.^14^ A previous study of human orthopedic and trauma surgery documented that the perforation detection rate by surgeons increased from 36–90% when indicator gloves were worn.^13^

The aims of this study were to investigate whether wearing inner colored indicator gloves would affect the perforation detection rate by surgeons and to determine the risk factors leading to an increased rate of glove perforation during veterinary orthopedic surgery.

## MATERIALS AND METHODS

This study was reviewed and approved by the Institutional Ethical Review Committee (VIN/14/024).

### Randomized Controlled Trial

The first part of the study was a prospective randomized controlled trial. The primary surgeon was 1 of 4 senior board‐certified surgeons or 3 surgical residents at Bristol University teaching hospital. Primary surgeons, and their assistant when present, double‐gloved for all procedures. A permuted block randomization procedure was performed with a block size of 10 to randomize whether the inner pair was a pair of standard surgical gloves (Biogel^®^ Surgeons, Molnlycke Healthcare, Bedfordshire, UK) or a pair of colored indicator gloves (Biogel Eclipse^®^ Indicator^®^ Underglove, Molnlycke Healthcare). None of the gloves used were specifically designed for orthopedic surgery. The surgeon was informed just before scrubbing which inner glove type to wear. The outer pair of gloves were standard surgical gloves for all surgeries. Perforations detected by surgeons during surgery were recorded and outer gloves were changed if requested by the surgeon. All gloves were leak‐tested after surgery according to a previously validated technique to identify perforations.^15^ Perforations were defined as any hole in outer or inner gloves documented by leakage of water. A planned interim analysis was performed after the first 180 procedures and a clear and significant difference in perforation detection with the use of indicator gloves was observed so this part of the study was terminated and only indicator gloves were used for the remaining procedures.

### Risk Factor Analysis

Small animal orthopedic procedures (n=300), defined as those primarily involving the musculoskeletal system, were included in the study over a period of 1 year (August 2014 to July 2015). Risk factors recorded included patient factors (age, sex, neutering status, breed, and bodyweight), type of surgery (fracture surgery, joint surgery, arthroscopic surgery, and others), surgical time, time surgery started, whether the primary surgeon was board‐certified (registered as a specialist with either the ACVS, ECVS, or RCVS), and the use of various orthopedic surgical instrumentation. Instruments included power tools (battery‐ or air‐powered), a saw (hand, battery‐, or air‐powered), hypodermic needles, orthopedic wire, arthrodesis/K‐wires, intramedullary Steinmann pins, interlocking nails, external skeletal fixators (ESF), and plate and/or screw fixation systems.

### Statistical Analysis

For the randomized controlled trial, the effect of indicator gloves on the surgeons' ability to detect glove perforations was assessed by Fisher's exact test. For the risk factor analysis, surgeries were binarized to whether a perforation had occurred or not. Continuous data were tested for a normal distribution using the Shapiro–Wilk test. Associations between glove perforation and potential risk factors were initially explored by univariate analysis using χ^2^, Fisher's exact, or Mann–Whitney U tests, or independent samples t‐test as appropriate. Factors associated with glove perforation in the univariate analysis (*P*<.10) were considered for inclusion in a multivariable logistic regression model using a forward stepwise approach. Factors were left in the model if the likelihood ratio test of comparing models with and without the factor showed evidence of a better fit with it (*P*<.05) and removed if *P*>.10. Factors were tested for collinearity and where significant interactions were detected (*P*<.05), only one variable was included in the model at any one time. Significance was set as *P*<.05. All statistics were performed using SPSS for Windows (version 23; IBM SPSS Statistics, Chicago, IL).

## RESULTS

### Incidence of Glove Perforation

**Figure 1:**
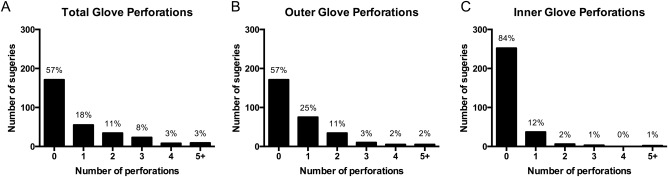
Frequency and number of glove perforations per procedure: (A) total perforations, (B) outer glove perforations, and (C) inner glove perforations.

In total, 574 double pairs of gloves worn during 300 procedures were collected over the study period. For 26 procedures there was no assistant surgeon present. One or more perforations were identified in 129 of 300 surgeries resulting in at least 1 perforation in 43% of procedures (Fig 1). A total of 293 perforations in 2,296 gloves were identified with 225 perforations in 1,148 outer gloves and 68 perforations in 1,148 inner gloves. The inner pair of gloves were not damaged in 81 of 129 (63%) surgical procedures where a glove perforation occurred, meaning an intact barrier was still in place between the animal and the surgeon's hand. Of the 293 perforations, 224 perforations (76%) occurred in gloves worn by the primary surgeon while 69 perforations (24%) occurred in the assistant's gloves. The primary surgeon sustained significantly more perforations in their gloves than their assistants (*P*<.001). Of the 293 perforations, the number of perforations affecting the nondominant hand (171; 58%) was not significantly different from the dominant hand (122; 42%; *P*=.32).

### Randomized Controlled Trial to Assess the Effect of Indicator Gloves on Glove Perforation Detection

**Figure 2:**
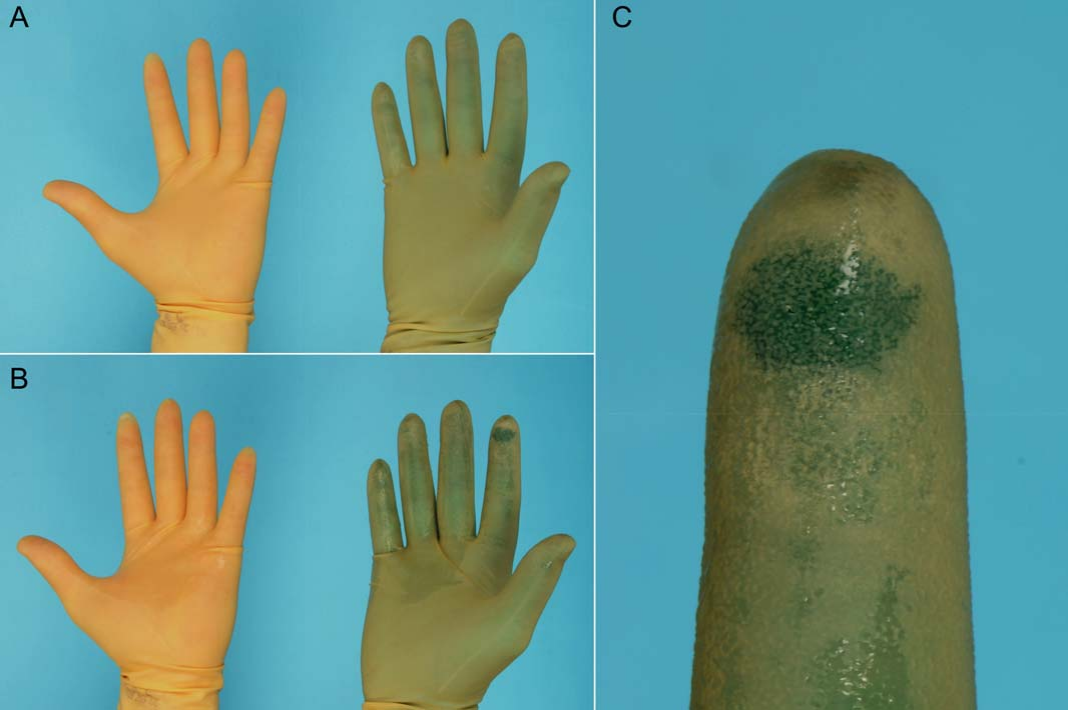
(A) Double gloving with standard (left) and colored indicator gloves (right). (B) A perforation was created in the index finger using a 23 gauge hypodermic needle, which shows a colored spot when indicator gloves are worn (right) and which is not visible when 2 standard gloves are worn (left). (C) Enlarged view of the colored spot indicating perforation has occurred.

One double pair of gloves included at least 4 gloves worn by a single surgeon (left and right inner and outer gloves). There were 95 double pairs of gloves worn by surgeons where ≥1 perforations occurred (26%). Of these 95 double pairs of gloves, when 2 pairs of standard gloves were worn, 41 double pairs contained ≥1 perforation (23%), and of these the perforation was detected in 14 (34%). When indicator gloves were worn as the inner glove, 54 double pairs of gloves contained ≥1 perforation (30%) and of these the perforation was detected during surgery in 45 (83%). Perforations were significantly more likely to be detected when indicator gloves were worn (*P*<.001; Fig 2).

### Risk Factor Analysis of Factors Associated with Glove Perforation

All significant risk factors reported increased the likelihood of glove perforation except for arthroscopy, which was protective against the risk of perforation (Table 1). In the multivariate analysis, the independent risk factors that remained in the final model were use of plates and/or screws, placement of an ESF, use of orthopedic wire, and the primary surgeon being board‐certified (Table 2). No significant interactions between these 4 parameters were detected. Perforations occurred in 50% of the 170 surgeries involving the use of plates and/or screws. Perforations occurred in 75% of the 16 surgeries involving the placement of an ESF. Perforation occurred in 58% of 45 procedures during which an orthopedic wire was placed either as a cerclage or inter‐fragmentary wire. Perforation occurred during 191 of 300 (48%) of the surgeries in which a board‐certified surgeon was the primary surgeon compared to 35% of procedures when a resident was the primary surgeon.

**Table 1:** Factors that demonstrated association with glove perforation in the univariate risk factor analysis. All factors were significantly associated with glove perforation except arthroscopy, which was protective against perforation

Risk Factor	*P*‐Value	Statistical Test
Power tools	0.001	Fisher's Exact
Surgery time	0.005	Mann–Whitney U
Arthrodesis/K‐wire	0.007	Fisher's Exact
Plate/screws	0.007	Fisher's Exact
External skeletal fixator	0.010	Fisher's Exact
Board‐certified primary surgeon	0.030	Fisher's Exact
Orthopedic wire	0.035	Fisher's Exact
Fracture surgery	0.067	Fisher's Exact
Arthroscopy	0.072	Fisher's Exact

**Table 2:** Final multivariate risk factor analysis showing factors that were significantly independently associated with glove perforation in veterinary orthopedic surgery

Risk Factor	*P*‐Value	OR	95% CI
Plates/screws	.001	2.4	1.4‐4.0
External skeletal fixator	.002	7.0	2.1‐23.8
Orthopedic wire	.011	2.4	1.2‐4.7
Board‐certified primary surgeon	.016	1.9	1.1‐3.1

## DISCUSSION

Our randomized controlled trial demonstrates that wearing colored indicator gloves as the inner pair during double‐gloving increased the surgeon's ability to detect glove perforations from 34–83%. This finding is consistent with previous human surgical studies.^12^, ^13^, ^14^ The ability to detect holes quickly enables the surgeon to change their outer pair of gloves, which should hopefully reduce any potential contamination of the surgical site in the event that both pairs of gloves are perforated. Although hands are aseptically prepared before gloving, it is accepted that this will not completely eliminate commensal bacterial burden.^16^ For example, regular changing of the outer pair of gloves during human total hip arthroplasty surgery was required to result in a “sterile state” in 80% of cases.^17^ Although not quantified, surgeons in our study found changing the outer pair of double gloves far easier than replacing single gloves because of perspiration causing single gloves to stick to the skin. Because we were not investigating the effect of perforation on SSI, surgeons could change gloves at their discretion. In human surgery, glove changes are recommended as soon as perforations are identified and as a minimum every 90 minutes.^18^

Surgeons were asked to check their gloves carefully for evidence of perforation at the end of the procedure before removing them. Because of the nature of the study, it was not possible to blind surgeons to the glove study group as there was an obvious difference in glove color, which surgeons were able to observe during the procedure. This could have introduced some bias as the surgeons were aware of the hypothesis of the study—that glove perforations would be easier to detect using indicator gloves.

Overall, we documented a glove perforation rate of 43% during veterinary orthopedic procedures. This is consistent with a previous report in the veterinary literature of an overall perforation rate of 26.2%, which was almost doubled during orthopedic surgery in a different veterinary hospital.^6^ It is interesting that the reported incidence of perforation in human orthopedic and trauma surgery is lower (18.5%).^13^ Speculatively, this may reflect a higher degree of specialization within human surgery with increased familiarity with procedures, dexterity with instrumentation and implants, and consequently a lower perforation rate. There is also an increased concern within human surgery of surgeons contracting infectious diseases such as human immunodeficiency virus from infected patients, which may make surgeons naturally more cautious during surgery. Although it would seem prudent to attempt to limit glove perforations to maintain an aseptic barrier, studies investigating the contribution of glove perforation to SSI are still lacking in the veterinary literature.

In human orthopedic surgery, two pairs of surgical gloves are recommended to provide an additional barrier between surgeon and patient. Single gloves are 4 times more likely to be perforated during surgery compared to the inner pair of double gloves.^12^ To the authors' knowledge the use of double gloving has not previously been reported in a veterinary setting. In our study, the inner pair of gloves were intact in 63% of procedures where an outer glove perforation had occurred. This meant the surgical site was only exposed to the surgeon's hand in 16% of procedures because of inner glove perforation. This is lower than previously reported in veterinary orthopedic surgery where single gloves were worn.^6^, ^7^ There is a concern that double‐gloving will reduce surgeon dexterity and tactility compared to single‐gloving. However, a previous study of human surgeons observed no difference in dexterity or tactile sensation with double gloving compared to single gloving or not wearing gloves.^19^

In our study, the risk of glove perforation was increased in primary compared to assistant surgeons. This finding is logical and consistent with previously published data^6^ since the primary surgeon will be handling the instrumentation more than assistant surgeons. It is interesting, however, that there was no significant increase in perforation risk in the nondominant hand since this has previously been documented in both veterinary^7^ and human studies.^20^

From the multivariate analysis, the risk factors independently associated with glove perforation were found to be the use of plates and/or screws, placement of an ESF, use of orthopedic wire, and the primary surgeon being board‐certified. The use of intramedullary pins, arthrodesis/K‐wires, and the interlocking nail were not found to have an association with glove perforation. One of the previous veterinary studies investigating glove perforation also identified orthopedic wire to be significantly associated with glove perforation,^6^ but did not investigate the additional risk factors identified in our study. The finding that board‐certified primary surgeons were more likely to perforate their gloves than residents was unexpected and may be attributable to the more complex procedures they are likely to perform compared to residents. However, a previous veterinary study did not find any clear relationship between surgical experience and glove perforation rate.^7^

There were several limitations to our study. We assessed 20 different risk factors in our univariate analysis, which is relatively high for the 300 cases included in the study. Although we were able to demonstrate significance for independent risk factors in the multivariate analysis, inclusion of additional cases may have allowed detection of additional significant risk factors that did not remain in our final multivariate model. It would have been interesting to also investigate the effect of glove perforation on SSI. However, inner gloves were only perforated in 48 cases and, given the rate of SSI for veterinary orthopedic surgery is reported to range from 0.5–1.3%,^9^ our study was not sufficiently powered to determine the effect of perforation on SSI. In addition, our study was performed at a single veterinary hospital and results may therefore not be transferable to other institutions.

In conclusion, we report that wearing indicator gloves as the inner pair of gloves during surgery increases the detection of perforations by surgeons during orthopedic surgery, enabling damaged gloves to be changed expediently ensuring an aseptic barrier is maintained. Independent risk factors for glove perforation during orthopedic surgery were the use of plates and/or screws, placement of an ESF or orthopedic wire, and the primary surgeon being board‐certified. We advocate double‐gloving, with indicator gloves as the inner pair, for orthopedic surgeries, particularly when using plates and/or screws or orthopedic wire, or when placing an ESF.

## DISCLOSURE

The authors declare no conflicts of interest related to this report.
